# Identification of Children With Autism Spectrum Disorder Based on Multidimensional EEG Feature Fusion Across Temporal-Spectral-Spatial Domains

**DOI:** 10.31083/AP45250

**Published:** 2026-04-27

**Authors:** Jiannan Kang, Liang Zhang, Xiaoke Yang, Xiaoli Li, Xiwang Fan, Shukai Zheng

**Affiliations:** ^1^College of Electronic & Information Engineering, Hebei University, 071002 Baoding, Hebei, China; ^2^State Key Laboratory of Cognitive Neuroscience and Learning, Beijing Normal University, 100875 Beijing, China; ^3^Clinical Research Center for Mental Disorders, Shanghai Pudong New Area Mental Health Center, School of Medicine, Tongji University, 200092 Shanghai, China

**Keywords:** autism spectrum disorder, electroencephalography, machine learning, neural pathways, support vector machine

## Abstract

**Background:**

To better characterize the complex neural features of autism spectrum disorder (ASD) and overcome the limitations of traditional electroencephalography (EEG) analysis methods, we developed a multi-metric EEG framework integrating temporal, spectral, and spatial dimensions, systematically characterized the dynamics, individualization, and nonlinear network features of neural oscillations in ASD, and evaluated their classification performance.

**Methods:**

Children with ASD (*n* = 44) and typically developing (TD) children (*n* = 44) were recruited and resting-state EEG data were collected. The analysis was conducted from three perspectives: (1) temporal domain — Lempel-Ziv complexity (LZC) was used to quantify the dynamic complexity of signals; (2) frequency domain — the gedBounds method based on generalized eigen decomposition (GED) was applied to identify individualized frequency bands; (3) spatial domain — Generalized Symbolic Nonlinear Granger Causality (GSNGC) was used to construct brain functional networks and compute graph-theoretic metrics. Finally, a support vector machine (SVM) integrated multidimensional features for ASD classification.

**Results:**

In the temporal domain, the ASD group showed significantly lower whole-brain LZC compared with the TD group, with the most pronounced reduction observed in the alpha band, suggesting reduced neural dynamic information processing capacity. In the frequency domain, the ASD group showed an expanded theta bandwidth, reduced low-frequency power in central-occipital regions, and increased beta power in frontal regions. In the spatial domain, children with ASD exhibited an atypical connectivity pattern characterized by increased low-frequency connectivity, reduced alpha-band connectivity, and increased beta-band connectivity, along with significantly higher global efficiency in theta and beta networks. The SVM model integrating temporal, frequency, and spatial features achieved an accuracy of 89.2%, significantly outperforming single-domain feature models, confirming that multidimensional feature integration improves classification performance.

**Conclusions:**

This study introduces a novel analytical approach combining individualized frequency band identification, nonlinear connectivity modeling, and dynamic complexity analysis. The findings comprehensively reveal multi-scale abnormalities of neural oscillations in children with ASD and demonstrate the discriminative power of multi-dimensional EEG feature integration for ASD classification and auxiliary diagnosis, thereby providing a scientific basis for clinical diagnosis and intervention.

**Clinical Trial Registration:**

No: ChiCTR2400092790. 24 November, 2024, https://www.chictr.org.cn/showproj.html?proj=249950.

## Main Points

1. Children with autism spectrum disorder (ASD) exhibited reduced neural dynamic complexity (Lempel-Ziv complexity) in the alpha band and atypical frequency bandwidths, particularly in the theta band. These abnormalities were observed across various regions, including frontal, central, and occipital regions, indicating a diminished capacity for neural dynamic information processing in ASD.

2. The individualized frequency band identification approach, using generalized eigen decomposition (GED), revealed that ASD children have significantly altered spectral characteristics, such as broader theta bandwidth and lower stability of frequency bands, compared to typically developing children.

3. The study found that children with ASD showed atypical brain functional networks, including enhanced low-frequency connectivity and reduced alpha-band connectivity, as well as hyperconnectivity in the beta band. These patterns were linked to key ASD symptoms, such as sensory hypersensitivity and impaired cognitive flexibility.

4. This study introduces a novel multi-metric electroencephalography (EEG) framework that integrates temporal, spectral, and spatial dimensions to better characterize the neural oscillations in children with ASD. The framework demonstrates the enhanced classification performance of ASD, with an accuracy of 89.2% using support vector machine (SVM) classification.

## 1. Introduction

Autism spectrum disorder (ASD) is a neurodevelopmental disorder that manifests in early childhood, with core symptoms including social deficits, impaired language communication, and repetitive, stereotyped behaviors [1]. The current global prevalence has approached 1% and continues to rise [2]. Neuroimaging studies have shown that children with ASD exhibit widespread brain functional abnormalities, including enhanced functional connectivity within the default mode network (DMN) [3] and impaired integration of the frontoparietal control network (FPN) [4]. However, the limited temporal resolution of fMRI prevents the capture of millisecond-level neural dynamics.

Electroencephalography (EEG), with its advantage of high temporal resolution, provides an important avenue for investigating neural oscillations and their temporal dynamics in ASD. EEG studies have reported significant abnormalities in the complexity and rhythmic stability of brain signals in children with ASD, such as reduced complexity and disrupted rhythmicity [5, 6, 7]. In addition, a recent EEG microstate study demonstrated that the peak frequency of the alpha rhythm in children with ASD shows high heterogeneity, with variability significantly greater than that of typically developing (TD) children, reflecting marked abnormalities in neural oscillatory activity [8]. Regarding functional connectivity, EEG analyses have shown that children with ASD exhibit a significantly higher proportion of time in strongly connected states within the alpha band, whereas TD children show a more balanced distribution across different connectivity states [9]. Such atypical connectivity patterns may constitute a neurophysiological basis closely associated with the core symptoms of ASD.

However, traditional resting-state power spectral analyses struggle to capture the dynamic modulation of brain rhythms during tasks. Specifically, individuals with ASD demonstrate significantly impaired dynamic reorganization of alpha rhythms during perception and attention tasks, changes that are often obscured by time-averaged processes that result in loss of dynamic information [10]. In addition, fixed-frequency-band partitioning often overlooks the frequency shift phenomenon commonly observed in individuals with ASD [11]. Approximately 40% of ASD individuals show an alpha peak frequency shifted toward the lower-frequency range of 7–10 Hz. Such enhanced low-frequency alpha activity may compromise the accuracy of power estimations and lead to systematic misinterpretations of neural oscillatory activity [9, 12]. Common linear connectivity metrics, such as phase-locking value (PLV) and coherence, primarily rely on linear-coherence analysis and are inadequate for identifying sparse and nonlinear network structures frequently observed in ASD. Studies have indicated that under sparse connectivity conditions, such metrics are highly susceptible to noise interference and fail to effectively integrate complementary information across networks. The false positive rate can reach up to 25%, substantially undermining the interpretability of connectivity patterns [13, 14, 15].

Although numerous studies have investigated EEG in ASD children, the substantial heterogeneity and individual variability within ASD make it challenging for traditional single-dimensional EEG methods to comprehensively characterize the dynamic features of neural oscillations, individual spectral differences, and nonlinear network structures in ASD [16, 17].

Several other studies have also used multi-dimensional EEG features for ASD classification. Dey *et al*. achieved promising classification performance in multivariate machine-learning tasks using features such as power spectra and functional connectivity [18]. Aslam, by integrating temporal- and frequency-domain EEG features with an LSTM autoencoder [19], achieved a classification accuracy of 93% in ASD diagnosis. Those studies provided methodological insights for the present work.

To address this gap, we developed a multi-metric EEG framework that integrates temporal, spectral, and spatial dimensions to characterize neural oscillations in ASD through their dynamics, individualization, and nonlinearity. For the temporal domain, the present study introduced Lempel-Ziv complexity (LZC) to quantify the instantaneous complexity of EEG signals. LZC reduces signal complexity through binarization, making the signals more regular and predictable, which may account for the sharp oscillations and focal spikes observed in children with ASD, suggesting potential biomarker value [20]. In the frequency domain, the gedBounds method based on generalized eigen decomposition (GED) was used to achieve adaptive frequency-band partitioning according to individual peak frequencies, thereby enhancing the sensitivity of spectral analysis at the individual level [21]. In the spatial domain, symbolic nonlinear Granger causality (GSNGC) was applied; it integrates nonlinear analysis, noise robustness, and statistical validation, allowing for more accurate extraction of brain-network functional connectivity patterns from noisy data and revealing nonlinear functional connections between brain regions [22, 23].

By integrating individualized frequency-band identification, nonlinear-connectivity modeling, and dynamic-complexity analysis, this study presented a novel systematic framework for ASD EEG analysis. We assessed the ASD classification performance of the three integrated EEG feature types using a Support Vector Machine (SVM) model, thereby exploring their value for practical clinical use [24].

## 2. Materials and Methods

### 2.1 Participants

A total of 44 children with ASD (mean age = 5.52 years, SD = 1.78) and 44 age-matched typically developing (TD) children (mean age = 5.81 years, SD = 1.73) were recruited. No significant differences were observed between the two groups in terms of age (t (86) = 0.15, *p* = 0.873, Cohen’s d = 0.03) or sex distribution (χ^2^ (1) = 0.08, *p* = 0.768, Cramer’s V = 0.03) (see Table 1).

**Table 1.** **Demographic characteristics of the participants**.

Variable	ASD Group (n = 44)	TD Group (n = 44)	Statistic (ASD vs. TD)	*p*-value
Age (years)	5.52 ± 1.76	5.79 ± 1.71	t (86) = 0.15	0.873
Gender (M/F)	37/7	38/6	χ^2^ (1) = 0.08	0.768

ASD, Autism Spectrum Disorder; TD, Typically Developing; M, male; F, female.

The inclusion criteria for children with ASD were as follows: (a) a diagnosis of ASD made by a qualified child psychiatrist according to the Diagnostic and Statistical Manual of Mental Disorders, Fifth Edition (DSM-5); (b) aged between 4 and 6 years; and (c) written informed consent obtained from their legal guardians.

The exclusion criteria were as follows: (a) a history of neurological disorders, such as epilepsy, brain injury, or having undergone neurosurgery; and (b) use of antipsychotic or anticonvulsant medications during or prior to the study period.

In this study, the ASD children had limited language abilities and reduced self-care skills, and could only understand and follow simple instructions; therefore, the ASD participants in this study were all classified as low-functioning individuals.

The inclusion criteria for TD children were as follows: (a) aged between 4 and 6 years with normal intelligence, and (b) written informed consent provided by their guardians. Some participating TD children underwent intellectual assessments, with Intelligence Quotient (IQ) scores ranging from 90 to 105, which fell within the normal range.

### 2.2 Data Acquisition and Preprocessing

All resting-state EEG data were collected in a quiet laboratory with a comfortable ambient temperature. Children were accompanied by their parents and seated in a comfortable chair, while the experimenters provided simple and clear instructions to help them remain relaxed with eyes open. During data acquisition, the children’s behavioral state was monitored in real time to ensure data quality. An eight-channel EEG recording system (model JL-EEG-8, Jielian Medical, Jiangxi, China) was used, with electrodes placed at F3, F4, T3, C3, C4, T4, O1, and O2. All electrode impedances were maintained below 20 kΩ, with Cz used as the reference electrode. The data were sampled at 1000 Hz, and the recording duration for each child was approximately 5 min.

Offline preprocessing was performed using MATLAB R2020a software (https://www.mathworks.com) in conjunction with the EEGLAB v2022.0 toolbox (https://eeglab.org). The raw continuous EEG data were first visually inspected to identify channels with severe noise or instability, which were later restored through interpolation at the end of preprocessing. Considering that artifacts may be introduced by electromagnetic interference, power-line noise, participant movements, electrocardiographic, and ocular activities, the preprocessing pipeline included the following steps: A zero-phase narrowband notch filter centered at 50 Hz was first applied to suppress power-line interference, followed by a zero-phase FIR band-pass filter of 0.5–45 Hz to retain the primary physiological frequency bands. Subsequently, an integrated approach combining ensemble empirical mode decomposition and independent component analysis (EEMD-ICA) was applied to remove artifacts such as eye movements, blinks, and electromyographic activity [25], with a mean of 1.6 independent components removed per dataset. Residual artifact segments were further excluded through visual inspection to ensure data quality. All EEG signals were then re-referenced to the average reference. The final duration of EEG data retained for each child was three min, with no significant differences between groups in terms of preprocessed data length. The data were subsequently segmented into 2-s epochs for further analysis.

### 2.3 EEG Temporal-Complexity Analysis

We quantified the temporal complexity of EEG signals using the LZC algorithm [26]. LZC is a widely used nonlinear dynamic analysis metric that evaluates the structural diversity and informational richness of signals in the temporal domain. It reflects the brain’s information processing capacity and functional flexibility, which are often reduced in neurodevelopmental disorders such as ASD [27, 28].

Preprocessed EEG signals were segmented into non-overlapping 4-s epochs, consistent with the approach used in previous EEG complexity studies. Each epoch was binarized using the median threshold method, converting the continuous time series into a binary (0-1) sequence suitable for LZC calculation [29]. LZC was computed for each channel and epoch, and the results were subsequently averaged across all epochs to obtain the overall LZC metric for each participant.

### 2.4 Individualized Frequency-Band Analysis of EEG

Traditional EEG spectral analysis typically uses standardized, fixed-frequency-band boundaries (e.g., the alpha band is uniformly defined as 8–13 Hz). However, substantial evidence has indicated that the peak frequency of neural oscillations, particularly the alpha rhythm, varies considerably across individuals, with this variability being especially pronounced in children with ASD [8, 11]. Using fixed frequency bands may misalign individual oscillatory activity, leading to inaccurate power spectral estimates. To address this, the present study used the gedBounds method [30], which is based on Generalized Eigen Decomposition (GED), to automatically extract individualized frequency-band boundaries from multi-channel EEG signals. By maximizing the separability between the signal and reference covariance matrices, this method identifies frequency features from spatially coherent oscillatory activities. The processing pipeline is as follows:

#### 2.4.1 Construction of Covariance Matrices

The continuous EEG data were segmented into 2-s epochs, and a covariance matrix was computed for each epoch. To exclude unrepresentative segments, the Euclidean distance between covariance matrices and the average covariance matrix across all epochs was calculated and then standardized using z-score normalization. Only epochs with distances within 3 standard deviations were retained to reconstruct a more representative average covariance matrix [31].

*Narrowband-signal-matrix construction:* The raw EEG data were band-pass filtered at target frequency points ranging from 2 to 100 Hz in 0.5 Hz steps to obtain a channels-by-time matrix $X_{f}$.

*Broadband-signal-matrix construction:* The original unfiltered data were used to obtain a channels-by-time matrix $X_{b}$.

Based on the above matrices, two covariance matrices were calculated:

(1) $$S=n^{-1}\,X_{f}\,X_{f}^{T},R=n^{-1}\,X_{b}\,X_{b}^{T}$$

Where n is the number of time points, S and R represent the signal covariance matrix and the reference covariance matrix.

#### 2.4.2 Solving the Optimal Spatial Filter

To obtain a spatial filter _w_ that best distinguishes between narrowband and broadband activity, the Rayleigh entropy needs to be maximized:

(2) $${\text{w}}_{\text{max}}=\arg\max_{\text{w}}\frac{{\text{w}}^{T}\,\text{Sw}}{{\text{w}}^{T}\,\text{Rw}}$$

Generalized eigen-decomposition of S and R to solve the eigenvalue problem:

(3) SW=RW⁢Λ

Where Λ is the eigenvalue and W is the eigenvector. This process is repeated for each frequency point to obtain a set of eigenvectors used for subsequent frequency band boundary identification.

#### 2.4.3 Eigenvector Clustering and Frequency-Boundary Identification

All eigenvectors across frequency points were paired to calculate squared correlation coefficients, thereby constructing a frequency-similarity matrix. A density-based clustering algorithm (e.g., DBSCAN) was then applied in order to cluster frequency points with high similarity. Each cluster represents a functionally coherent frequency band with consistent spatial patterns, enabling the identification of individualized frequency boundaries [30].

#### 2.4.4 Pseudocode

The pseudocode for this section is presented below (Algorithm 1).

**Table:** 

**Algorithm 1** gedBounds Individualized Frequency Band Partitioning
1: Input: Preprocessed multi-channel electroencephalography (EEG) data *EEG*_data_, Frequency Range: 1–30 Hz; step = 0.5 Hz
2: Output: List of individualized frequency band boundaries freq_bands_
3: # Preprocessing and Segmentation
4: Apply band-pass filtering (1–30 Hz) to *EEG*_data_ and segment into 2s epochs
5: Compute covariance matrix for each epoch
6: Remove outlier epochs (Euclidean distance from average covariance matrix >3 standard deviations)
7: Compute clean average covariance matrix *R*
8: # Generalized Eigenvalue Decomposition (GED) Loop
9: **for** *f* in Frequency Range **do** (e.g., from 1 to 30 Hz in 0.5 Hz steps) # Iterate over target frequencies
10: Apply narrow-band filtering to *EEG*_data_ at frequency *f*
11: Compute average covariance matrix *S* of the narrow-band filtered data
12: Solve generalized eigenvalue problem: *S* · *W* = *λ* · *R* · *W*
13: Store the largest eigenvalue *λ_max_(f)* and corresponding eigenvector *W(f)*
14: **end for**
15: # Frequency Band Boundary Identification
16: Smooth the maximum eigenvalue spectrum *λ_max_(f)* across all frequency points *f*
17: Identify significant peaks in the *λ_max_(f)* spectrum
18: Determine frequency band boundaries freq_bands_ at the troughs between two peaks, or based on predefined rules
19: **return** freq_bands_

### 2.5 EEG Spatial Analysis Based on GSNGC Functional Connectivity

We introduced the Genuine Symbolic Nonlinear Granger Causality (GSNGC) framework [32] to assess functional connectivity in children with ASD. This method integrates symbolic dynamics, nonlinear causality analysis, and surrogate testing to eliminate spurious connections and enhance the noise robustness of EEG signals.

The preprocessed EEG signals were used to generate surrogate time series through the Iterative Amplitude Adjusted Fourier Transform (IAAFT) algorithm [33]. For each channel, 20 surrogate sequences were generated, preserving the amplitude distribution and power spectrum of the original data while randomizing the phase information.

Both the original and surrogate signals were transformed into symbolic sequences [34]. For a time series $x=\left\{x_{t}\right\},t=1,2,\ldots ,n$, the state space was reconstructed using an embedding dimension of m = 4 and a time delay τ (optimized based on the dataset; see Parameter Selection) as follows:

(4) $$X_{t}=\left[\begin{array}{c} x_{t},x_{t-\tau },x_{t-2\,\tau },\ldots ,x_{t+\left(m-1\right)\,\tau } \end{array}\right]$$

The selection of τ was based on both empirical ranges and experimental validation: first, candidate τ values were set within the reasonable range of 1–3 according to the literature; then, with the embedding dimension fixed at m = 4, data segment length L = 2000, and 80% overlap, the effect of different τ values on the GSNGC network’s ability to capture group-level statistical differences was systematically tested. Finally, τ = 2 was chosen as the optimal parameter, as it most clearly and stably distinguished functional networks between the ASD and TD groups.

Ordinal patterns were generated by sorting the elements within the reconstructed vectors in ascending order, converting the continuous EEG signals into symbolic sequences (e.g., $x⟶x_{\mathrm{sym}}$).

The directed causal strength between pairs of symbolic sequences $\left(x_{\text{sym}},y_{\text{sym}}\right)$ was quantified using kernel-based nonlinear Granger causality [35].

(5) $${\text{Nonlinear GC}}_{x\to y}=\ln\left(\frac{{\overset{`}{o}}_{y}}{{\overset{`}{o}}_{y|x}}\right)$$

where ${\overset{`}{o}}_{y}$ and ${\overset{`}{o}}_{\mathrm{y|x}}$ represent the prediction errors of $x_{\text{sym}}$ without and with the historical information of $x_{\text{m}}$, respectively. A Gaussian kernel (δ = 4) was used to map the symbolic sequences into a reproducing kernel Hilbert space (RKHS).

To eliminate false positive connections, a Wilcoxon signed-rank test (*p* < 0.001) was applied to compare the original connectivity (Nonlinear $G\,C_{\text{original}}$) with the null distribution generated from surrogate data (Nonlinear $G\,C_{\text{surr}}$) [36]. Effective connections were required to meet the following criteria.

(6) $$G\,S\,N\,G\,C=\left\{ \begin{array}{cc} G\,S\,N\,G\,C_{\text{original}} & :H=1,p<0.001\\ 0 & :\text{otherwise} \end{array}\right.$$

Based on the functional-connectivity matrices extracted using the GSNGC method, brain networks were constructed, and topological features were further calculated to investigate the functional patterns of brain networks in ASD. For each participant, the GSNGC connectivity matrix was treated as a weighted functional-connectivity graph, with nodes corresponding to EEG channels and edge weights representing significant functional-connectivity strengths. Subsequently, the Brain Connectivity Toolbox [37] was used to extract the following graph-theoretical metrics to quantify the topological characteristics of the brain networks:

*Clustering coefficient (CC)*: represents the density of connections surrounding each node.

(7) $$C\,C=\frac{1}{N}\,\sum_{i=1}^{N}\frac{2\times e_{i}}{k_{i}\times \left(k_{i}-1\right)}$$

*Characteristic path length (CPL)*: reflects the shortest paths between any two nodes within the network.

(8) $$C\,P\,L=\frac{1}{N\,\left(N-1\right)}\,\sum_{i\neq j}d_{i\,j}$$

*Global Efficiency (GE)*: Measures the information integration capability of the entire network.

(9) $$G\,E=\frac{1}{N\,\left(N-1\right)}\,\sum_{i\neq j}\frac{1}{d_{i\,j}}$$

*Local Efficiency (LE)*: Measures the efficiency of information integration and transmission within a node’s neighborhood, reflecting the fault tolerance of the local network.

(10) $$L\,E=\frac{1}{N}\,\sum_{i=1}^{N}\left(\frac{1}{k_{i}\,\left(k_{i}-1\right)}\,\sum_{j,k\in N_{i}}\frac{1}{d_{j\,k}^{\left(i\right)}}\right)$$

Here, $e_{i}$ is the actual number of edges between the neighbors of node i, $k_{i}$ is the degree of node i (the number of its neighbors). *N* is the total number of nodes in the network. $d_{\text{ij}}$ is the shortest path length from node i to node j. dd is the shortest path length between nodes i and j after removing node i.

The pseudocode for the GSNGC part is shown below (see Algorithm 2):

**Table:** 

**Algorithm 2** GSNGC Analysis Pipeline		
1: Input: Two time series x,y; embedding dimension m = 4; delay τ = 3; Gaussian kernel width σ = 4; number of surrogate datasets n = 20; significance level α = 0.001.
2: Output: Surrogate-corrected, noise-resistant nonlinear Granger causality value Genuine Symbolic Nonlinear Granger Causality (GSNGC).
3: # Symbolization:
4: a. Reconstruct state space for x and y (embedding dimension m, delay τ).
5: b. Convert each state vector into a symbolic sequence (based on the ordinal pattern of its values).
6: Compute original GSNGC: Calculate the nonlinear Granger causality value GSNGC_original_ from x_sym_ to y_sym_ in the Reproducing Kernel Hilbert Space (RKHS) using the Gaussian kernel σ.
7: # Generate Null Distribution:
8: a. **for** i = 1 **to** n **do**
9: i. Generate surrogate series y_sym_ preserving linear properties using the Iterative Amplitude Adjusted Fourier Transform (IAAFT) algorithm.
10: ii. Repeat steps 4–5 for y_sym_, computing GC(i)surr.
11: **end for**
12: # Statistical Testing: Use the nonparametric Wilcoxon signed-rank test to compare GSNGC_original_ with the surrogate distribution $\left[{\text{GC}}_{\text{surr}}\right]$, obtaining a *p*-value.
13: # Output:
14: **if** *p*-value < α **then**
15: GSNGC = GSNGC_original_ (Significant connection exists)
16: **else**
17: GSNGC = 0 (Connection not significant, set to zero)
18: **end if**
19: **return** GSNGC

### 2.6 Statistical Analysis

All statistical analyses were performed using MATLAB R2020b software. Prior to analysis, the Kolmogorov–Smirnov test was used to assess data normality. For non-normally distributed data, a logarithmic transformation was applied; if normality was still not met, non-parametric tests (such as the Mann–Whitney U test or Wilcoxon signed-rank test) were used. Baseline differences between the ASD and TD groups were assessed using independent samples *t*-tests (or non-parametric tests). All results were reported after false discovery rate (FDR) correction to control for multiple comparisons. For significant group differences, Cohen’s *d* was also calculated as a measure of effect size, providing a quantitative assessment of the practical significance of between-group differences.

## 3. Results

### 3.1 EEG-Complexity-Analysis (LZC) Results

**Fig. 1.**
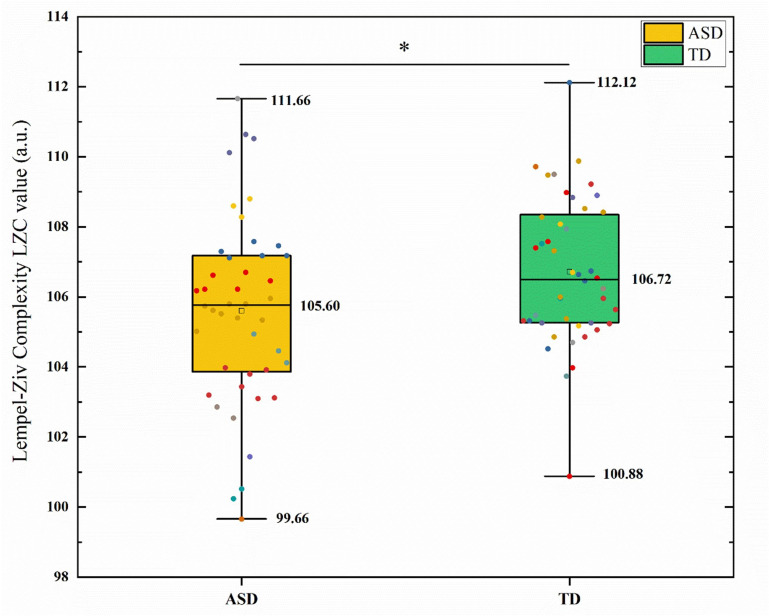
**Comparison of global lempel-ziv complexity (LZC) between the autism spectrum disorder (ASD) and typically developing (TD) groups**. The box plot shows the distribution of LZC values, with the ASD group significantly lower than the TD group. * indicates a significant difference after false discovery rate (FDR) correction (*p* < 0.05).

**Fig. 2.**
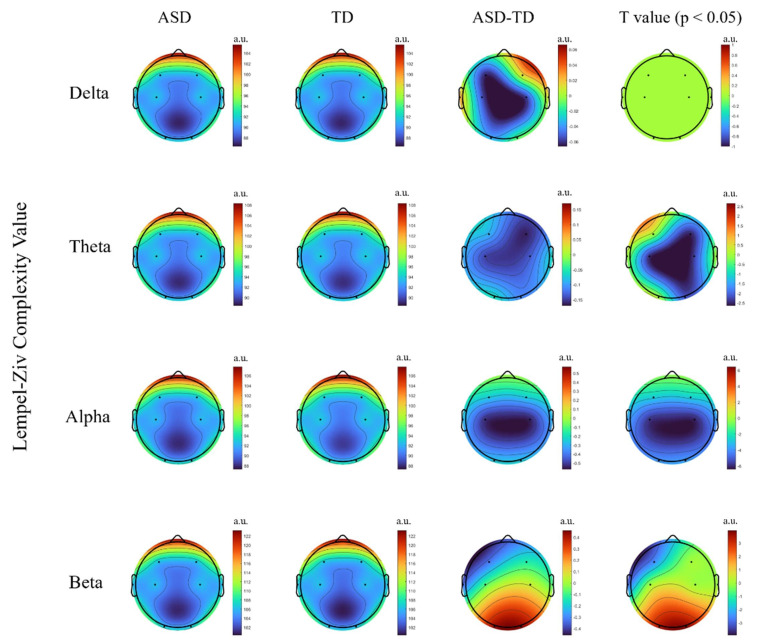
**Group comparison of lempel-ziv complexity (LZC) between the autism spectrum disorder (ASD) and typically developing (TD) groups across different frequency bands**. The figure shows the differences in LZC values between groups in the main brain regions for the Delta, Theta, Alpha, and Beta bands. t-values surviving false discovery rate (FDR) correction at *p* < 0.05 are displayed.

The present study compared the temporal complexity LZC of resting-state EEG signals between the ASD and TD groups using independent-samples *t*-tests. Significant *t*-test results were further corrected for multiple comparisons using FDR. The results are shown in Fig. 1. The global LZC values showed that the ASD group was significantly lower than the TD group (t = –4.42, *p* < 0.001, Cohen’s *d* = 0.94). In the band-specific analysis (see Fig. 2), no significant difference was observed in the delta band between the two groups (t = –1.12, *p* = 0.267, Cohen’s *d* = 0.94). In the theta band, the ASD group exhibited significantly lower LZC than did the TD group in the frontal-central regions (F4, C3, C4; t = –2.35, *p* = 0.021, Cohen’s *d* = 0.51). In the alpha band, LZC was significantly reduced across the whole brain in the ASD group (t = –4.07, *p* = 0.002, Cohen’s *d* = 0.87). In the beta band, the frontal regions (F3, F4) also showed significant reductions in LZC (t = –3.79, *p* = 0.005, Cohen’s *d* = 0.81), whereas in the parietal–occipital regions, the ASD group exhibited significantly lower LZC (t = 2.89, *p* = 0.031, Cohen’s *d* = 0.62).

### 3.2 Individualized Band Power Analysis (gedBounds) Results

**Fig. 3.**
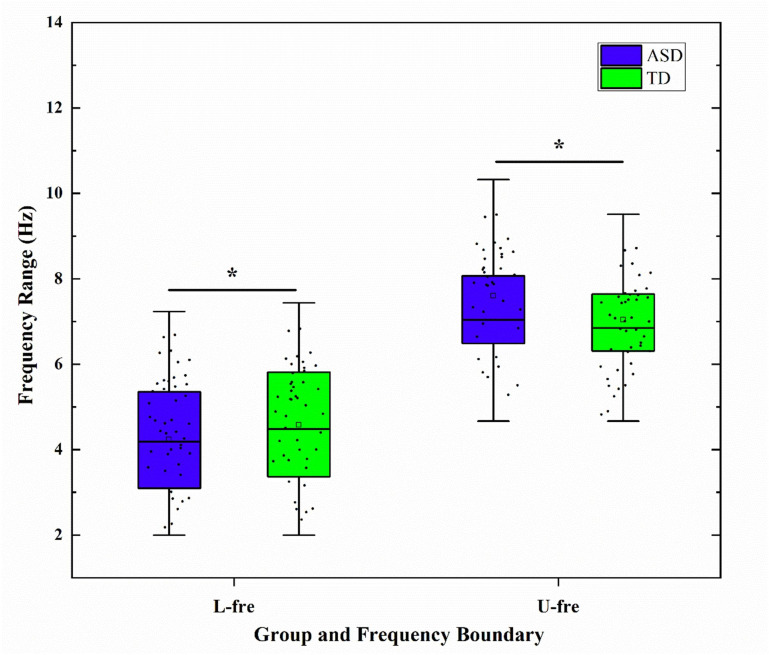
**Boxplots of group differences in the lower and upper bounds of the Theta band between autism spectrum disorder (ASD) and typically developing (TD) groups**. L-fre, Lower frequency limit; U-fre, Upper frequency limit; * indicates a significant difference after false discovery rate (FDR) correction (*p* < 0.05).

The GED method was used to determine the individualized frequency bands based on the peak spectral curves (see Table 2). The results showed that children with ASD had a significantly lower lower-frequency limit in the theta band, a wider band range, and less stable boundaries; this was significantly different from children with TD (see Fig. 3). Although the re-test reliability of the band boundaries was not directly assessed in this study, it has been shown that eigenvalue spectral decomposition-based band delineation has good stability across time points [38] (Intraclass Correlation Coefficient, ICC >0.8), supporting its reproducibility basis as a tool for individualized spectral analysis.

**Table 2.** **Comparison of individualized frequency boundaries between autism spectrum disorder (ASD) and typically developing (TD), showing the distribution of lower and upper bounds of the individualized frequency bands**.

Frequency range	Group	Delta (Hz)	Theta (Hz)	Alpha (Hz)	Beta (Hz)
Lower frequency limit	ASD	2.18 ± 0.30	4.18 ± 1.47	8.55 ± 2.17	14.98 ± 4.14
	TD	2.13 ± 0.27	4.57 ± 1.46	8.81 ± 2.28	14.94 ± 3.78
Upper frequency limit	ASD	3.79 ± 1.02	7.60 ± 1.82	12.47 ± 1.66	29.36 ± 2.28
	TD	3.82 ± 1.03	7.04 ± 1.33	12.55 ± 1.76	29.51 ± 1.77

**Fig. 4.**
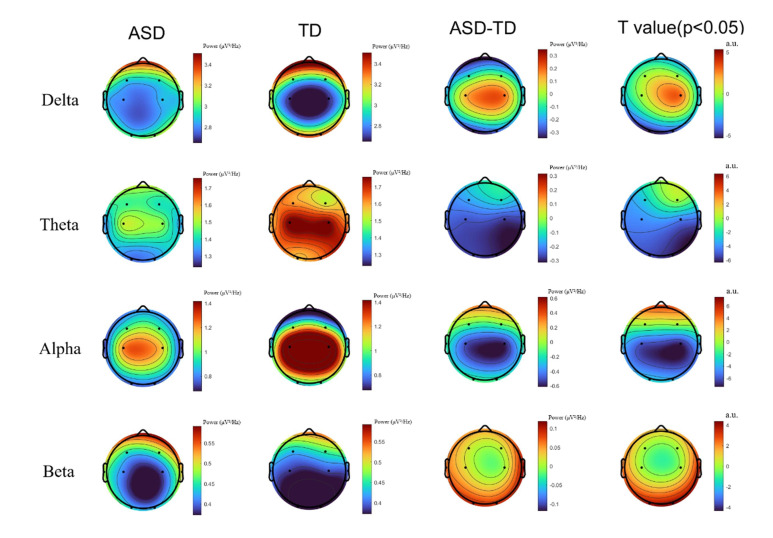
**Comparison of power spectral topographies between autism spectrum disorder (ASD) and typically developing (TD) children across the Delta, Theta, Alpha, and Beta frequency bands**. t-values after false discovery rate (FDR) correction at *p* < 0.05 are displayed.

After applying FDR correction to the significant results in the low-frequency bands (delta and theta), TD children exhibited significantly higher individualized power than did children with ASD. These differences were primarily distributed from the central to occipital regions, particularly around C3, C4, and O1. ASD children exhibited an overall reduction in power in the alpha band, whereas TD children showed higher alpha power in the occipital region. In contrast, in the high-frequency beta band, ASD children demonstrated significantly higher power than did TD children in the prefrontal and some central regions (see Fig. 4).

### 3.3 Symbolic Granger Causality (GSNGC) Results

**Fig. 5.**
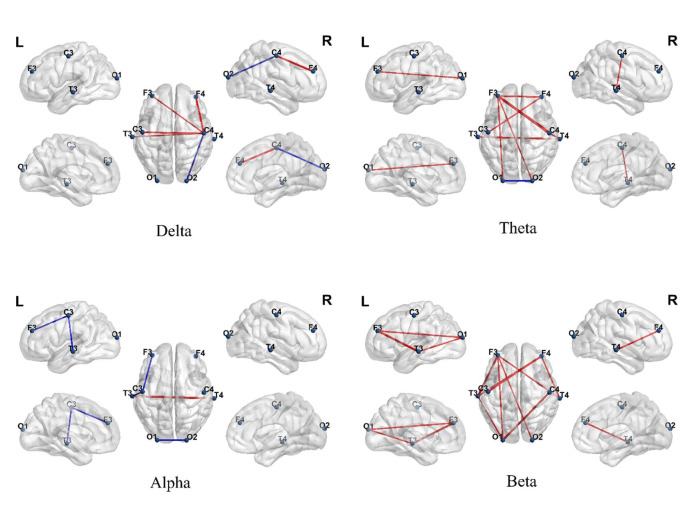
**Differences in brain functional connectivity between autism spectrum disorder (ASD) and typically developing (TD) groups across different frequency bands**. Each subplot shows significant connectivity differences extracted by generalized symbolic nonlinear Granger causality (GSNGC) in the Delta, Theta, Alpha, and Beta bands. Red lines indicate connections stronger in the ASD group; blue lines indicate connections stronger in the TD group (false discovery rate, FDR correction *p* < 0.05). L, left; R, right.

Significant functional-connectivity differences emerged between the ASD and TD groups across multiple frequency bands (Fig. 5). Within the delta band, the ASD group exhibited significantly enhanced functional connectivity, primarily concentrated from the frontal to central regions (e.g., F3, F4 to C3, C4) and from central to occipital areas (e.g., O1, O2). In the theta band, the ASD group showed stronger connectivity from the prefrontal to parieto-occipital regions (e.g., F3–O1, F3–O2), whereas the TD group displayed stronger connectivity only in a few posterior pathways (e.g., O1–O2). In the alpha band, the TD group demonstrated stronger functional connectivity across multiple central and occipital pathways (e.g., C3–T3, O1–O2), whereas the ASD group generally showed weakened connectivity. In the beta band, the ASD group again exhibited stronger connectivity, covering the frontal, central, and occipital regions (e.g., F3–C4, C4–T4, O1–O2). These results suggested differences in resting-state functional connectivity patterns across multiple frequency bands and brain regions between ASD and TD children.

### 3.4 Network-Characteristics-Analysis Results

**Fig. 6.**
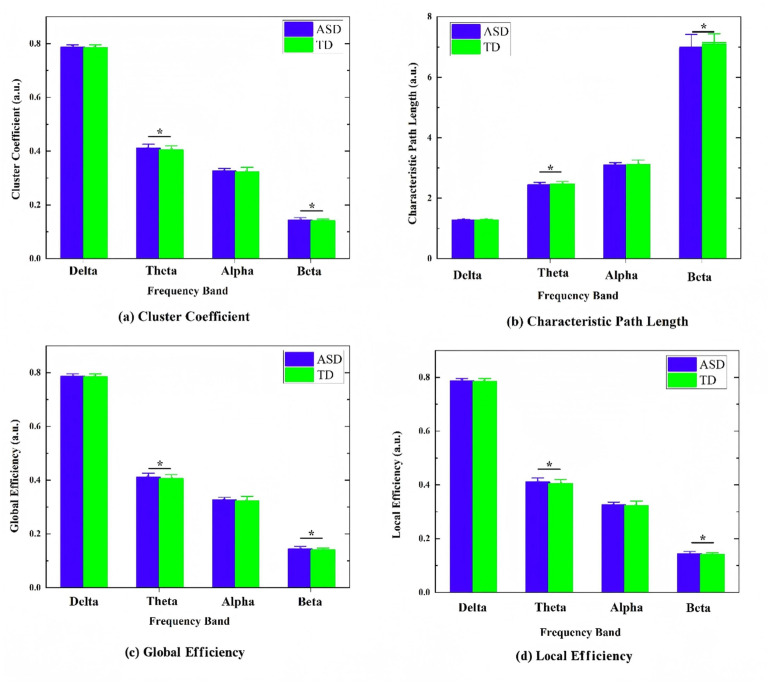
**Comparison of brain-network graph-theory metrics between autism spectrum disorder (ASD) and typically developing (TD) groups across different frequency bands (Delta, Theta, Alpha, Beta)**. The figure shows the distribution of common graph metrics for each band: (a) clustering coefficient, (b) characteristic path length, (c) global efficiency, and (d) local efficiency, * indicates a significant difference after false discovery rate (FDR) correction (*p* < 0.05).

Network analysis further revealed distinct topological alterations in the ASD group, primarily within the theta and beta bands (Fig. 6). In the theta band, the ASD group showed a significantly higher clustering coefficient (t = 2.25, *p* = 0.025), global efficiency (t = 2.67, *p* = 0.008), and local efficiency (t = 2.42, *p* = 0.016) than did the TD group, although the characteristic path length (t = –2.05, *p* = 0.041) in the ASD group was slightly shorter than that of the TD group. In the high-frequency beta band, multiple network metrics including clustering coefficient (t = 2.51, *p* = 0.013), global efficiency (t = 3.942, *p* < 0.001), and local efficiency (t = 2.41, *p* = 0.017) were significantly higher in the ASD group than in the TD group. No consistent significant differences were observed in the delta and alpha bands.

### 3.5 ASD Classification Results Based on Integrated Temporal, Spectral, and Spatial Features

To evaluate the diagnostic efficacy of multi-dimensional EEG features in ASD classification, we constructed an SVM classification model based on integrated temporal (4 × 8), frequency (4 × 8), and spatial (8 × 7 × 4 + 8 × 4 × 4) features. A radial basis function (RBF) was used as the kernel for the SVM, with a penalty parameter C = 1. Although other hyperparameters were not optimized, the model still achieved relatively good classification performance.

A subject-level 5-repetition 10-fold cross-validation was employed, in which all epoch-level features from each participant were aggregated for analysis. This approach ensured complete independence between the training and testing sets, effectively preventing data leakage. The model achieved an average accuracy of 89.21% (95% CI: 85.1%–92.8%), a sensitivity of 87.54% (95% CI: 82.3%–91.9%), a specificity of 89.65% (95% CI: 85.0%–93.2%), an F1-score of 89.72% (95% CI: 85.5%–93.2%), and an area under the curve (AUC) of 0.91 (95% CI: 0.90%–0.97%), demonstrating robust classification performance. Further comparison of classification performance across different feature dimensions showed that using LZC features alone yielded an accuracy of 64.62% (±4.2%), individualized frequency-band power alone achieved 76.11% (±5.1%), and functional connectivity alone reached 81.67% (±4.8%) (see Table 3). After integrating all three feature types, accuracy increased significantly, indicating that multi-dimensional feature-fusion enhanced the discriminative capability of the model. For comparison, a baseline model using only age and sex achieved an accuracy of 52.6% (95% CI: 41.2%–63.1%) and an AUC of 0.51. All EEG feature–based models significantly outperformed the baseline model, indicating that EEG features played a critical role in distinguishing children with ASD from TD children.

**Table 3.** **Comparison of model performance with different feature combinations**.

Feature combination	Accuracy (%)	Sensitivity (%)	Specificity (%)	F1-Score (%)	AUC
T	64.62	66.31	67.25	63.57	0.67
F	76.11	75.85	77.36	75.24	0.78
S	81.67	80.57	82.34	83.31	0.84
T + F	82.51	81.43	83.91	82.21	0.85
T + F + S	89.21	87.54	89.65	89.72	0.91

T, Temporal domain; F, Spectral domain; S, Spatial domain. T + F indicates a combination of temporal and spectral features; T + F + S represents the combined features of all three domains.

### 3.6 Sensitivity Analysis

To verify the robustness of our findings, a sensitivity analysis was conducted. All male participants were selected from the original sample (ASD: *n* = 37; TD: *n* = 38), and the SVM modeling and evaluation were performed using the same multi-dimensional EEG features and classification procedures as in the full-sample analysis. The results showed that, even within the male-only sample, the integrated model (T + F + S) still achieved an accuracy of 87.9% (±4.3%), comparable to the performance observed in the full sample (89.2%).

## 4. Discussion

The present study systematically compared the EEG characteristics of ASD and TD children across three dimensions: temporal complexity, individualized spectral features, and spatial-network topology. The results revealed significant abnormalities in neural dynamics, oscillatory patterns, and functional-connectivity structures in the ASD group, providing neurophysiological evidence for brain dysfunction in ASD. The SVM classification results confirmed the ability of multidimensional EEG indicators to differentiate ASD from TD, demonstrating their value in characterizing neural mechanisms and supporting clinical diagnosis.

We observed significantly reduced LZC in the ASD group, which indicated less dynamic variability in neural signals and less flexible information encoding in this group than in TD children. Lower LZC indicated that the brain activity patterns in children with ASD were more stereotyped and less diverse, which may be closely related to behavioral characteristics such as restricted adaptive behaviors, reduced cognitive flexibility, and difficulty adapting to novel situations [39, 40]. Our study found a significant decrease in LZC in the alpha band. This finding aligns with the characteristics of increased neural rigidity and reduced whole-brain dynamic state transition frequency reported by Watanabe and Yamasue [41]. Such reduced neural dynamics reflected impaired information processing flexibility in individuals with ASD, supporting the core hypothesis of “diminished neural dynamics in ASD” and providing evidence at the dynamic-systems level for understanding limitations in information processing associated with ASD.

The spectral-domain results showed that children with ASD exhibited systematic abnormalities in both the amplitude and frequency distribution of neural oscillations. The ASD group demonstrated a significantly broadened frequency bandwidth in the theta band, indicating decreased stability of low-frequency rhythms. Pagnotta *et al*. [42] confirmed that theta rhythms play a critical role in cognitive control within the frontoparietal network. Their study found that children with ASD exhibit significantly widened spectral bandwidth in the theta band, indicating impaired cross-frequency neural coordination. This mechanistic disruption further leads to diminished neural synchronization and reduced information-integration efficiency, providing support from the perspective of neural oscillation dynamics for Uhlhaas’s “neural oscillation coordination disorder” hypothesis [43].

Power spectral analysis further revealed frequency-band-specific abnormal patterns. In the low-frequency bands (delta and theta), power was significantly decreased in the central to occipital regions, consistent with Orekhova *et al*.’s findings [44] in ASD children of thalamo-cortical pathway dysfunction related to arousal regulation and sensory gating. The significant reduction of alpha-band power in the occipital region supported the presence of deficits in visual integration and attentional inhibition in ASD [45]. This may lead to sensory hypersensitivity in children with ASD, making it difficult for them to filter irrelevant information, thereby affecting perceptual stability and social interaction abilities [46]. Studies have indicated that the beta band is closely associated with executive function, motor planning, and cortical excitation-inhibition balance [47, 48]. The increased beta power in the prefrontal-central regions observed in children with ASD indicated hyperactivation of the sensorimotor network and potential excitation-inhibition imbalance, which may contribute to behavioral rigidity and limited cognitive flexibility in ASD.

In the spatial-connectivity dimension, functional connectivity networks constructed based on GSNGC revealed significant differences between ASD and TD children across multiple frequency bands, reflecting atypical organization patterns of resting-state brain networks. Specifically, in the delta and theta bands, children with ASD showed widespread enhanced low-frequency connectivity from the frontal to central and occipital regions, which may represent fundamental neural regulatory imbalances or compensatory activation mechanisms [49]. In the alpha band, TD children exhibited stronger connectivity in central and occipital pathways (e.g., C3–T3, O1–O2), whereas the ASD group showed overall weakened connectivity, indicating reduced functional integration in pathways related to perceptual processing and intrinsic attention regulation [50]. In the beta band, connectivity was enhanced in ASD children, and involved frontal, central, and occipital regions, which are potentially linked to abnormal hyperactivation of sensorimotor networks associated with repetitive behaviors and executive dysfunction [51]. Overall, children with ASD showed a distinct brain-connectivity pattern: enhanced low-frequency connections, abnormal high-frequency activity, and weakened connections in specific bands. This pattern suggested that their neural impairments may stem from poor coordination between brain rhythms and reduced ability to integrate information across brain networks [52, 53].

Further network-characteristic analysis revealed that in the theta and beta bands, the ASD group exhibited significantly shorter CPL than did the TD group, indicating impairments in both global information integration and local connectivity organization. The topological changes in the theta band may be associated with neural deficits in cognitive control and executive function, whereas the decreased network efficiency in the alpha band likely reflects reduced information-processing capacity related to perceptual processing and social interaction [54, 55].

Combined spectral and spatial cross-dimensional analysis revealed coordination dysregulation of neural oscillations across multiple frequency bands in children with ASD. The significant broadening of the theta bandwidth may disrupt the theta–gamma phase-amplitude coupling mechanism, leading to excessive enhancement of theta connectivity in the frontal regions (e.g., F3–C3). This is consistent with the fMRI findings of Zhou *et al*. [56] regarding compensatory activation. The increased beta band power in the frontal area, together with hyperconnectivity in sensorimotor networks, jointly points to excitation/inhibition (E/I) imbalance mechanisms in ASD [57].

The SVM classification model constructed in this study achieved an accuracy of 89.2% after integrating temporal, frequency, and spatial features. In comparison, Heinsfeld *et al*. [58], using the ABIDE dataset and convolutional neural networks (CNNs) for automatic feature extraction, achieved only approximately 70% accuracy. Moreover, the classification task in the present study, based on integrated features, significantly outperformed any single-domain model and other classification approaches. This performance improvement stemmed from the complementary nature of the three-dimensional features in neural representation: the temporal domain reflects dynamic complexity; the spectral domain characterizes oscillatory stability; and the spatial domain captures functional integration capacity. Together, they synergistically depict the multi-scale neural dysfunctions in ASD. Compared to single metrics that may overlook the heterogeneity of pathological mechanisms, the integrated model provided a systematic quantification of whole-brain functional impairments in ASD.

### Limitations

Despite providing valuable preliminary findings, this study had several limitations. First, due to the limited number of EEG channels, the spatial resolution was relatively low, making it difficult to resolve neural activity in deep brain regions precisely. Future studies should combine high-density EEG or fMRI to provide complementary validation. Second, the sample in this study was restricted to children aged 4–6 years, which limited the generalizability of the findings. Subsequent research should include a broader age range to characterize the developmental trajectory of neural dynamics in ASD. Third, this study did not systematically quantify the relationship between EEG features and core clinical symptoms of ASD. Future work could integrate behavioral scales (e.g., Social Responsiveness Scale, SRS, Autism Behavior Checklist, ABC) to perform correlation and modeling analyses. Finally, before translation into clinical tools, the findings must be validated in larger, multi-center independent cohorts. Future studies should recruit more representative samples to examine the generalizability of multi-dimensional EEG features.

## 5. Conclusions

The present study used a multimodal EEG-analysis approach to systematically compare the brain electrical characteristics of ASD children and TD children using three dimensions: temporal complexity, individualized spectral features, and spatial network topology. The results revealed significant abnormalities in neural dynamics, oscillatory patterns, and functional connectivity structures in children with ASD. The support-vector-machine classification model integrating temporal, spectral, and spatial multidimensional features demonstrated high diagnostic accuracy, providing new insights and methods for studying the neural mechanisms of ASD and aiding clinical diagnosis.

## Availability of Data and Materials

The datasets generated and analyzed in this study are available from the corresponding authors upon reasonable request.
